# Research advances in mechanism of antiangiogenic therapy combined with immune checkpoint inhibitors for treatment of non-small cell lung cancer

**DOI:** 10.3389/fimmu.2023.1265865

**Published:** 2023-10-16

**Authors:** Danming He, Lu Wang, Jiachen Xu, Jie Zhao, Hua Bai, Jie Wang

**Affiliations:** Chinese Academy of Medical Sciences (CAMS) Key Laboratory of Translational Research on Lung Cancer, State Key Laboratory of Molecular Oncology, Department of Medical Oncology, National Cancer Center/National Clinical Research Center for Cancer/Cancer Hospital, Chinese Academy of Medical Sciences & Peking Union Medical College, Beijing, China

**Keywords:** lung cancer, immunology, anti-angiogeneic therapy, research advance, immune checkpoint inhibitors

## Abstract

Immunotherapy has changed the treatment strategy of non-small cell lung cancer (NSCLC) in recent years, among which anti-PD-1/PD-L1 antibodies are the most used. However, the majority of patients with NSCLC do not derive benefit from immune checkpoint inhibitors (ICIs). Vascular abnormalities are a hallmark of most solid tumors and facilitate immune evasion. Thus, combining antiangiogenic therapies might increase the effectiveness of anti-PD-1/PD-L1 antibodies. In this paper, the mechanisms of anti-angiogenic agents combined with anti-PD-1/PD-L1 antibodies are illustrated, moreover, relevant clinical studies and predictive immunotherapeutic biomarkers are summarized and analyzed, in order to provide more treatment options for NSCLC patients.

## 1 Introduction

Lung cancer is a disease that seriously endangers human health, and it also remains the leading cause of cancer death, including non-small cell lung cancer (NSCLC) and small cell lung cancer (SCLC) (1). NSCLC accounts for 80% - 85% of all lung cancers (2). In recent years, accurate treatment and combined therapies of lung cancer have made rapid progress, including immune checkpoint inhibitors (ICIs), ICIs combined with cytotoxic chemotherapies (Chemo), ICIs combined with ICIs and combined with anti-angiogenic therapy (3). Anti-PD-1/PD-L1 antibodies are the most used among ICIs (4). Additionally, the effects of ICIs + anti-angiogenic therapy are considerable, with accepted adverse events (AEs) (5). The results of a series of related clinical trials have been announced, which have been summarized here. Tumor angiogenesis provides nutrition for tumor growth, while lymphocytes infiltrate into the tumor through tumor vessels, and vascular growth interacts with tumor growth and tumor microenvironment (TME) to form a complex tumor ecological environment (6), so it is necessary to systematically review it. From this perspective, we described the mechanisms and summarized the latest progress of related clinical research of anti-angiogenic drugs combined with immunotherapies in NSCLC.

## 2 Crosstalk between tumor angiogenesis and immune microenvironment

### 2.1 Inhibiting effect of tumor angiogenic factors on tumor immune microenvironment

Abnormal vasculature is the hallmark of solid tumors, and is also involved in tumor immune escape (7). The abnormal vessels and impaired perfusion can also restrict the entry of cytotoxic drugs and immune cells from the circulation into tumors, limiting their anticancer activity. The TME consists of numerous pro-angiogenic factors, including vascular endothelial growth factor (VEGF), fibroblast growth factor (FGF), and platelet-derived growth factor (PDGF), which are secreted by tumor cells or tumor-infiltrating lymphocytes (TILs) or macrophages (8). These factors can activate pro-angiogenic signaling pathways to promote growth, invasion, and metastasis of tumor (9). Through the production of various cytokines and growth factors, such as VEGF, angiopoietin 2 (ANG-2), the immune cells work in concert with tumor cells lining abnormal tumor blood vessels to promote tumor angiogenesis and immunosuppression.

VEGF has suppressive effects on tumor immune microenvironment, by directly affect the differentiation, infiltration and cytotoxicity of various types of immune cells and other indirect mechanisms (10). VEGF could inhibit the differentiation of hematopoietic stem cells into CD4+ and CD8+ T cells in thymus. VEGF promotes the recruitment and proliferation of immunosuppressive cells such as regulatory T cells (Tregs), bone myeloid-derived suppressor cells (MDSCs), and M2-like tumor-associated macrophages (TAMs) (11). M2-like macrophages are similar in phenotype to TAMs, which promote tumor growth and metastasis, and are associated with poor prognosis of tumors (12). In addition, VEGF inhibits T cell proliferation and cytotoxicity, binding to VEGFR2 on T cell, and also up-regulates immune checkpoint molecules such as the programmed cell death protein 1 (PD-1), programmed cell death ligand 1 (PD-L1) and cytotoxic T lymphocyte-associated protein 4 (CTLA-4) to inhibit T cell activation (13). VEGF also can reduce the ability of immune cells to adhere and pass through the blood vessel, by down-regulating the integrin ligands intercellular adhesion molecule 1 (ICAM-1) and vascular cell adhesion protein 1 (VCAM-1) of endothelial cells or preventing them from accumulating on endothelial cells, thus prevent immune cells from entering the tumor (14). Dendritic cells (DCs) play a crucial role in T cell activation. However, VEGF-VEGFR2 signaling inhibits antigen presenting by interfering with DCs maturation, thus indirectly inhibiting T cell activity and resulting in decreased T cell-mediated anti-cancer activity (15). Moreover, VEGF could indirectly affect the biochemical properties of TME and promotes angiogenesis that results in an aberrant tumor vasculature, leading to hypoxia and a low pH in the TME, which in turn fosters immunosuppression both locally and systemically (16, 17).

**Figure 1:**
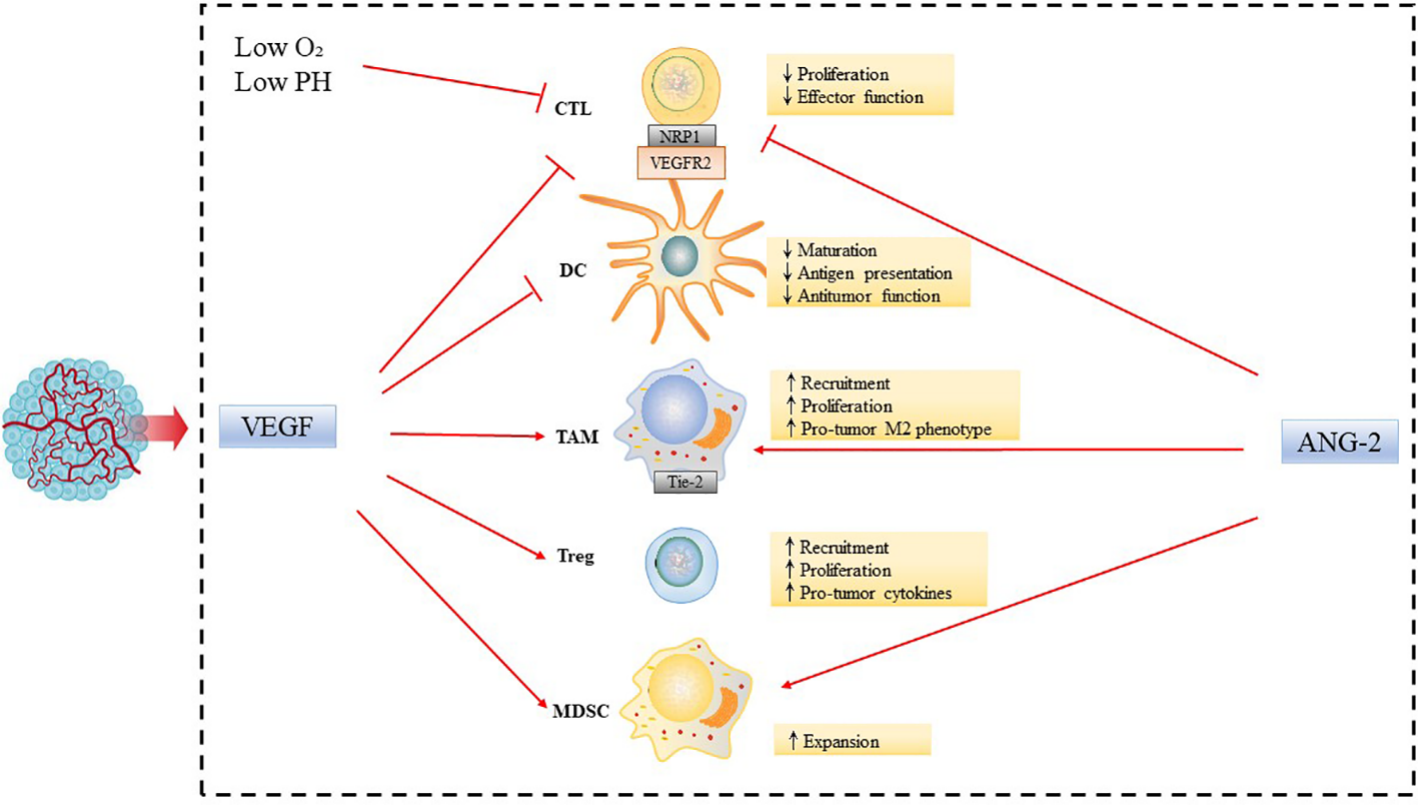
Inhibiting effect of tumor angiogenic factors on tumor immune microenvironment. Abnormalities in the tumor vasculature result in hypoxia and acidosis of the TME. Tumor angiogenic factors, such as VEGF and ANG-2, modulate the functions of immune cells leading to tumor immunosuppressive microenvironment, by increasing accumulation, activation, and expansion of Tregs; recruitment of inflammatory monocytes and TAMs and reprogramming of TAMs from an anticancer M1-like phenotype towards the pro-tumor M2 phenotype; suppression of DC maturation, which results in impaired antigen presentation and activation of CTLs, and expansion of MDSCs.

In addition to VEGF, ANG-2 is another key vascular growth immunomodulator. Activated ANG-2 signals can induce immunosuppressive TME through a variety of mechanisms (18). ANG-2 binds to Tie-2 expressed on monocytes, which recruits monocytes and also stimulates monocytes to secrete IL-10. IL-10 suppresses CD8+ cytotoxic T lymphocytes (CTLs) proliferation and cytotoxicity, and enhances Tregs infiltration (19). Moreover, ANG-2 also inhibits the secretion of transforming growth factor α (TNF-α), thereby limiting the anti-cancer activity of monocytes (18) (**Figure 1**). Overall, abnormalities in cancer blood vessels can cause immunosuppressive TME.

### 2.2 Promoting effect of tumor immune microenvironment on tumor angiogenesis

Tumor angiogenesis involves not only cancer cells but also a variety of immune cells, such as T cells, myeloid cell, and interstitial cell in TME. For the above immune cells, the release of proangiogenic cytokines is accompanied by a switch to an immunosuppressive behavior. T cells do not directly secrete VEGF, but they facilitate its effect by acquiring neuropilin 1 (NRP1) during interaction with DCs, which binds to VEGFA to promote angiogenesis (20). Both neutrophils and TAMs promote angiogenesis by secreting proangiogenic factors, such as VEGF, TNF-α, IL-8, and various chemokines including CXCR-2, 4 and 12, CXCL-3, 4, 8, 9, 10, and CCL2-5 (21). MDSC recruitment to the tumor can be induced by many different factors, such as CSF-3, IL-1β, and IL-6, and subsequently lead to activation of STAT3, rendering them potent as proangiogenic and immunosuppressive cells (22). Besides, Tregs indirectly inhibit tumor angiogenesis by inhibiting helper T cells (TH1) cells, which express IFN-γ (23). In short, immune cells in TME can promote tumor angiogenesis.

## 3 The mechanism of antiangiogenic drugs combined with immunotherapy

**Figure 2:**
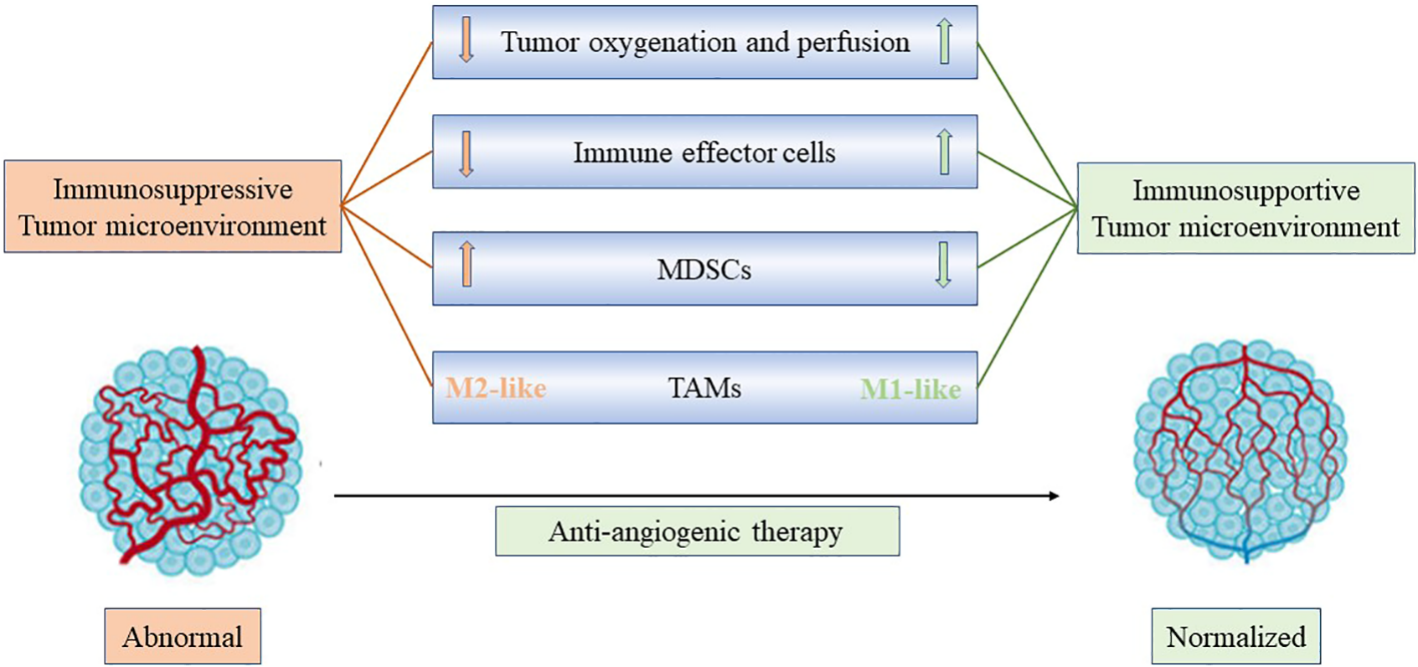
Anti-angiogenic treatment reprograms the tumor microenvironment from immunosuppressive to immunosupportive and improves effects of immunotherapy. Anti-angiogenic drugs induced vascular normalization improves immune cell infiltration and promotes the transformation of “cold tumors” into “hot tumors”, facilitating the infiltration of T effector cells while reducing MDSC accumulation. In addition, improved vascular perfusion polarizes TAMs to an immune stimulatory M1-like phenotype. Consequently, through anti-angiogenic treatment, vascular normalization could potentially enhance the effectiveness of immunotherapy.

Anti-angiogenic drugs induced vascular normalization improve immune cell infiltration and promote the transformation of “cold tumors” into “hot tumors”, thus enhances the efficacy of immunotherapy (24, 25). Anti-angiogenic drugs promote immune cell maturation and improve infiltration by blocking the binding of VEGF to VEGFR2 on the surface of macrophages and T cells (26). Tumor vascular normalization relieves hypoxia and reduces the secretion of VEGF, thus reducing the recruitment of immunosuppressive cells such as MDSCs and Tregs, and also reduce the expression of PD-1, PD-L1, CTLA-4, TIM-3 and other immune checkpoint molecules on the surface of immunosuppressive cells (27, 28). Besides, the vascular normalization effect is associated with more efficient lymphocyte priming by antigen-presenting cells, TAM polarization to an M1-like phenotype, and accumulation of activated, IFN-γ expressing CD8+ T cells within the perivascular space (29, 30) M1-like TAMs are generally considered to be tumor-killing macrophages, primarily anti-tumor and immune-promoting (12) (**Figure 2**). In current clinical practice, the most used antiangiogenetic drugs include bevacizumab (targeting at VEGF-A), ramucirumab (targeting at VEGFR2) (31). Multiple therapeutic agents targeting VEGF and VEGF receptors have been developed and approved for use in cancers. Moreover, tyrosine kinase inhibitors (TKIs) can inhibit not only VEGFR but also FGFR, PDGFR and so on (32). The commonly used TKI drugs are lenvatinib, apatinib and anlotinib in NSCLC. Preclinical studies have proved that low-dose apatinib combined with PD-1/PD-L1 inhibitors can enhance its anti-tumor activity, and in the preliminary clinical application showed a good therapeutic effect (33).

For ICIs, these drugs have been thought to mainly affect T cells, which lead to activating T cells secret INF-γ which decreased endothelial VEGFA, and increased CXCL-9, CXCL-10 and CXCL-11, induced tumor vascular normalization (34, 35). Thus, vascular normalization in the setting of immune stimulation represents a novel mechanism for the antitumor effects of immune checkpoint blockade and provides a new understanding of tumor vascular remodeling and immune reprogramming. As for ICI, antibodies that block the interaction of PD-1 with its ligand PD-L1 and the binding of CTLA-4 to its receptor have been approved for clinical use (36). The former ones include atezolizumab and durvalumab, inhibiting PD-L1, and pembrolizumab, cemiplimab, nivolumab, camrelizumab, toripalimab, and sintilimab, inhibiting PD-1 receptors (**Table 1**).

**Table 1:** The approved indications of anti-PD-1/PD-L1 antibodies and anti-angiogenic drugs in the globe.

Agent/target	Indication	Approval
Nivolumab/anti–PD-1	SC NSCLC SCLC RCC HL HNC UC CRC HCC ESC MPM GC GEJC	2014-US 2015-EU 2018-PRC
Pembrolizumab/anti-PD-1	SC NSCLC SCLC RCC HL HNC UC CRC HCC ESC GC GEJC TNBC BC CC EC	2014-US 2015-EU 2018-PRC
Toripalimab/anti-PD-1	SC HNC UC	2108-PRC
Sintilimab/anti-PD-1	NSCLC HL HCC	2018-PRC
Camrelizumab/anti-PD-1	NSCLC HL HNC HCC ESC	2019-PRC
Durvalumab/anti-PD-L1	NSCLC SCLC BC	2017-US 2018-EU 2019-PRC
Atezolizumab/anti-PD-L1	SC NSCLC SCLC	2016-US 2017-EU 2020-PRC
Avelumab/anti-PD-L1	SC RCC UC	2017-US 2017-EU
Cemiplimab/anti-PD-L1	SC NSCLC	2018-US 2019-EU
Bevacizumab/anti-VEGFA	CRC MSCLC RCC BC CC	2004-US 2005-EU 2010-PRC
Ramuciruma/anti-VEGFR2	GC CRC NSCLC HCC	2014-US 2015-EU 2022-PRC
Lenvatinib	RCC HCC TC	2015-US 2015-EU 2018-PRC
Apatinib	GC ESC HCC NSCLC	2014-PRC
Anlotinib	NSCLC	2018-PRC

SC, skin cancer; NSCLC, non-small cell lung cancer; RCC, renal cell carcinoma; HL, Hodgkin lymphoma; HNC, head and neck cancer; UC, urothelial carcinoma; CRC, colorectal cancer; HCC, hepatocellular carcinoma; ESC, esophageal carcinoma; MPM, malignant pleural mesothelioma; GC, gastric cancer; GEJC, gastroesophageal junction cancer; TNBC, triple-negative breast cancer; BC, bladder cancer; CC, cervical cancer; EC, endometrial cancer; TC, thyroid carcinoma; EU, European Union; PRC, People’s Republic of China.

To sum up, anti-angiogenesis therapy normalizes tumor blood vessels and also improves tumor immune microenvironment. ICIs can activate T lymphocytes to secrete IFN- γ, to reduce local hypoxia and promote the normalization of tumor vessels, which demonstrates the synergistic effect of anti-PD-1/PD-L1 antibodies combined with antiangiogenic drugs and provides a theoretical basis for their combination in the treatment of NSCLC (37, 38).

## 4 Clinical practices of anti-PD-1/PD-L1 antibodies plus antiangiogenic therapy in NSCLC

Based on published clinical studies, generally, anti-PD-1/PD-L1 antibodies combined with antiangiogenetic therapy showed better efficacy than ICIs alone, and prolonged progression free survival (PFS) and overall survival (OS). Here, we focused only on NSCLC, and summarized current clinical studies on combination therapy of anti-PD-1/PD-L1 antibodies plus antiangiogenesis in patients according to different clinical care scenarios.

### 4.1 Advanced first-line therapy

According to NCCN guidelines V1.2023 NSCLC, one of the recommended first-line therapy for advanced adenocarcinoma, large cell, and NSCLC not otherwise specified (NOS) patients is carboplatin + paclitaxel + bevacizumab + atezolizumab (ABCP) (39), with PD-L1 expression whether ≥50% or ranges between 1%-49%. The recommendation was based on the result of IMpower150, an international, open label, phase 3 study in chemo-naive NSCLC patients. Compared with becacizumab plus chemotherapy (BCP) group, ABCP group had longer median PFS (mPFS) (8.3 months vs. 6.8 months; HR, 0.62; 95% CI, 0.52 to 0.74; P < 0.001) and median OS (19.2 months vs. 14.7 months; HR, 0.78; 95% CI, 0.64 to 0.96; P = 0.02) (40, 41). The combination of pembrolizumab and ramucirumab for first-line treatment was investigated in an expansion cohort of JDVF trial, and the objective response rate (ORR) was 42.3% (42). Another cohort in a three-arm prospective study (anlotinib combined with erlotinib, carboplatin plus pemetrexed/gemcitabine, and sintilimab) adopting anlotinib combined with sintilimab in untreated locally advanced/metastatic NSCLC patients indicated low incidences of adverse events and better clinical benefits than previous reports with ORR 72.7%, and mPFS 15.6 months (43). Other clinical studies and their results were briefly listed in **Table 2**, and most of these researches showed positive evidence.

**Table 2:** Clinical studies of anti-PD-1/PD-L1 antibodies plus antiangiogenetic therapy in NSCLC patients.

Research type	Year	Country/Race	Target	Combination regimen	Sample size (N)	Patients	Treatment line	Outcomes		
								PFS	OS	ORR
First-line treatment										
Phase III RCT IMpower150 (NCT02366143)	2018	Multiple	PD-L1&VEGF-A	Atezo + Beva + Carbo + Tax (40)	356	NSQ	1^st^	8.3 months	19.2 months	63.5%
Phase I trial JVDF (NCT02443324)	2021	Multiple	PD-1& VEGFR2	Pembro + Ramu (42, 44)	26	4SQ&22NSQ	1^st^	9.3 months	Not reached	42.3%
Phase Ib four-arm trial	2016	Japan	PD-1& VEGF-A	Nivo + Beva + Carbo + Tax (45)	6	NSQ	1^st^	Not reached	N	100%
Phase III RCT TASUKI-52	2021	Asian	PD-1& VEGF-A	Nivo + Beva + Carbo + Tax (46)	275	NSQ	1^st^	12.1 months	25.4 months	61.5%
Phase Ib/II trial (NCT03083041)	2022	China	PD-1& VEGFR2	Camre + Apa (47)	25	NSQ, hTMB, *EGFR/ALK-*	1^st^	9.6 months	Not reached	40.0%
Phase II single-arm trial	2022	Japan	PD-L1& VEGF-A	Atezo + Beva (48)	39	NSQ, hPD-L1, *EGFR/ALK/ROS1-*	1^st^	15.9 months	Not reached	64.1%
Three-arm trial (NCT03628521)	2022	China	PD-1&Multi	Sinti + Anlo (43)	22	12SQ&10NSQ, *EGFR/ALK/ROS1-*	1^st^	15.6 months	Not reached	72.7%
Phase II single-armtrial TELMA (NCT03836066)	2022	Spain	PD-L1& VEGF-A	Atezo + Beva (49)	38	NSQ, hTMB, *EGFR/ALK/ROS1-*	1^st^	13.0 months	Not reached	42.1%
Phase II single-arm trial LUN17-139	2022	Multiple	PD-L1& VEGF-A	Atezo + Beva + Carbo + Pemx (50)	30	NSQ	1^st^	11.3 months	22.4 months	42.9%
Later-line treatment										
Phase I trial	2014	USA	PD-1& VEGF-A	Nivo + Beva (51)	12	NSQ, Chemo-treated	2^nd^	37.1 weeks	Not reached	8%
Phase I trial JVDF (NCT02443324)	2019	Multiple	PD-1& VEGFR2	Pembro + Ramu (42, 44)	27	4SQ&23NSQ, Chemo-treated	≥2^nd^	9.7 months	26.2 months	30%
Phase Ib/II trial (NCT02501096)	2020	Multiple	PD-1&Multi	Pembro + Lenva (52)	21		≥2^nd^	5.9 months	N	33%
Phase Ia/b trial JVDJ (NCT02572687)	2020	Multiple	PD-L1& VEGFR2	Durva + Ramu (53)	28	7SQ&21NSQ, ICI/Ramu-naïve	≥2^nd^	2.7 months	11.0 months	11%
Phase Ib/II trial (NCT03083041)	2021	China	PD-1& VEGFR2	Camre + Apa (54)	105	NSQ, Chemo-treated, EGFR*/ALK-*	≥2^nd^	5.7 months	15.5 months	30.9%
Phase Ib trial	2021	China	PD-1&Multi	Camre + Anlo (55)	51	8SQ&43NSQ	≥2^nd^	8.2 months	12.7 months	13.3%
Perspective control study	2021	China	PD-1&Multi	Camre + Anlo (56)	44		3^rd^	N	N	TER 93.18%
Phase Ib trial (NCT03006887)	2022	Japan	PD-1&Multi	Pembro + Lenva (57)	3	Metastatic	≥2^nd^	3.4 months	N	0
Phase II single-arm trial	2022	USA	PD-L1& VEGFR2	Atezo + Ramu (58)	21	6SQ&15NSQ, Anti-PD-(L)1-treated	≥2^nd^	3.4 months	16.5 months	4.8%
Phase II RCT Lung-MAP S1800A (NCT03971474)	2022	Multiple	PD-1& VEGFR2	Pembo + Ramu (59)	69	28SQ&41NSQ, Anti-PD-(L)1&Chemo-resistant	≥2^nd^	4.5 months	14.5 months	22%
Phase II trial	2022	China	PD-1& VEGFR2	Camre + Apa (60)	25	SQ, ICI-naïve, Chemo-treated	2^nd^	6 months	13.3 months	32.0%
Phase II two-stage trial	2022	Korea	PD-L1& VEGF-A	Atezo + Beva (61)	24	1SQ&23NSQ, Chemo&Atezo-treated	≥3^rd^	5.6 months	14.0 months	12.5%
EGFR positive patients										
Phase II single-arm trial	2021	China	PD-L1& VEGF-A	Atezo + Beva + Carbo + Pemx (62)	40	NSQ, *EGFR-*TKI resistant	≥2^nd^	9.4 months	Not reached	62.5%
Phase Ib/II trial (NCT03083041)	2022	China	PD-1& VEGFR2	Camre + Apa (63)	43	2SQ&41NSQ, Chemo-treated, *EGFR/ALK*-TKI resistant	≥2^nd^	2.8 months	Not reached	18.6%
Phase III RCT ORIENT-31 (NCT03802240)	2022	China	PD-1& VEGF-A	Sinti + IBI305 + Cis + Pemx (64)	148	NSQ, *EGFR-*TKI resistant	≥2^nd^	6.9 months	Not reached	44%
Retrospective study										
Real-world retrospective study	2021	China	PD-1&Multi	Anti-PD-1 + Anlo (65)	62	29SQ&33NSQ	≥2^nd^	5.0 months	N	19.3%
Retrospective analysis	2021	China	PD-1&Multi	Anti-PD-1 + Anlo (66)	67	26SQ&41NSQ	≥2^nd^	6.9 months	14.5 months	28.4%
Retrospective analysis	2022	China	PD-1& VEGFR2	Camre + Apa + Radio (67)	53	Oligometastatic		N	N	79.2%

Atezo, atezolizumab; Beva, bevacizumab; Carbo, carboplatin; Tax, paclitaxel; Pembro, pembrolizumab; Ramu, ramucirumab; Nivo, nivolumab; (N)SQ, (non) squamous; Camre, camrelizumab; Apa, apatinib; hTMB, high TMB; hPD-L1, high PD-L1; Sinti, sintilimab; Anlo, anlotinib; Pemx, pemetrexed; Chemo, chemotherapy; N, not found; Lenva, lenvatinib; Durva, durvalumab; TER, total effective rate; Cis, cisplatin; Radio, radiotherapy.

However, in a meta-analysis that involved 8278 patients from 16 randomized controlled trials (RCTs) comparing efficacy and safety of different first-line immunotherapy combinations, including ABCP, pembrolizumab-chemo, atezolizumab-chemo, camrelizumab-chemo, tislelizumab-chemo, sintilimab-chemo, nivolumab-ipilimumab, nivolumab-ipilimumab-chemo, durvalumab-tremelimumab, and durva-tremelimumab-chemo, ABCP regimen showed best efficacy in PFS and ORR, but the advantages in OS and toxicity were not prominent (68). Similar findings were also revealed in a meta-analysis enrolling 19 phase II/III RCTs in which the ABCP regimen was ranked 5^th^ among all 17 regimens including ABCP, 5 ICI-monotherapy regimens, 7 ICI-Chemo regimens, 2 dual-ICI strategies, 1 dual-ICI-Chemo combination, and 1 Beva-Chemo regimen, in which overall OS, PFS, safety, and ORR were taken into consideration (69). Furthermore, a cost-effectiveness analysis from the perspective of the US health care sector showed that adopting pembrolizumab monotherapy when PD-L1≥50%, pembrolizumab combined with chemotherapy when PD-L1 between 1%-49%, and nivolumab plus ipilimumab when PD-L1<1% were more cost-effective treatment options, whose performance was better than ABCP (QALY 2.39) (70). Similar results were revealed in several other cost-effectiveness analyses (71). It should be emphasized that comparing treatment strategies across studies is challenging, though, clinicians should be cautious, prudent, and comprehensive when considering anti-PD-1/PD-L1 antibodies combined with antiangiogenic treatment, especially when ICI monotherapy or dual-ICIs strategies are available at the same time.

### 4.2 Advanced later line therapy

Compared with traditional chemotherapy, immunotherapy has achieved good benefits in patients with advanced NSCLC, but the benefits are lower for patients receiving second or higher line treatment (72). The resistance mechanisms are probably multifactorial, including tumor expressing low levels of PD-L1 and immunosuppressive TME. Based on the theoretical synergistic mechanism between vascular normalization and immune promotion in TME, the combination of antiangiogenetic agent and anti-PD-1/PD-L1 antibodies may be able to show surprising antitumor activity in the subsequent line treatment. Furthermore, the resistance mechanism in the late stage of targeted therapy remains not well revealed (73). And the persistent toxicities after initial treatment with cytotoxic chemotherapy not only cause adverse events but also affect the response to later line therapy (44). In the randomized phase II Lung-MAP substudy (S1800A), 136 patients with progression after previous treatment with ICI and platinum-based chemotherapy randomly received pembrolizumab plus ramucirumab or standard care, and the former group showed an improved OS (14.5 months vs. 11.6 months) (59). Phase Ia/b JVDF trial assessed the safety and antitumor activity of ramucirumab combined with pembrolizumab treatment in previously treated solid tumor patients (advanced gastric or gastro-esophageal junction adenocarcinoma, non-small-cell lung cancer, or urothelial carcinoma). There was one, seven, fifteen, and three of 27 NSCLC patients achieved CR, PR, SD, and PD, respectively, with ORR 30% and mPFS 9.7 months, which was a favorable result compared with anti-PD-1/PD-L1 antibody treatment alone in other studies (44). Most randomized phase III clinical trials using combined immunotherapy were designed for first-line therapy, and there are few data for NSCLC patients in second-line or higher settings. Further explorations for subsequent line treatment of advanced NSCLC patients with multi-line resistance were needed.

### 4.3 Perioperative therapy

Apart from locally advanced or metastatic NSCLC patients, whether patients in relatively early stages can benefit from the combination therapy is also worth exploring. When investigating the efficacy of the combination therapy, stage III patients are often excluded, and there was little related research focusing on perioperative NSCLC patients. Apart from efficacy, it is also important to emphasize perioperative safety, as the inhibition of angiogenesis might affect wound healing (74). As far as currently known, an interval of ≥4 weeks between drug administration and surgery is recommended (75). The major concerns of perioperative patients such as neoadjuvant efficacy, postoperative recurrence and metastasis, and wound bleeding and healing complications, have yet not been well studied. As a theoretical basis, a pre-clinical study found that the combination therapy of pembrolizumab and bevacizumab could transform tumors into an inflamed condition, thus inhibiting tumor growth and preventing postoperative recurrence and metastasis in a humanized neoadjuvant mouse model (75).

The clinical studies that have been published so far on perioperative patients are mainly case reports. Toripalimab + apatinib + pemetrexed + nedaplatin has been reported for preoperative induction therapy in a patient who achieved a PFS of 7 months (76). Nivolumab + anlotinib and atezolizumab + bevacizumab + chemotherapy have both been reported for postoperative adjuvant therapy and have shown clinically favorable responses (77, 78). These reports revealed the feasibility of anti-PD-1/PD-L1 antibodies plus antiangiogenesis combination therapy as either neoadjuvant or adjuvant therapy, but still further clinical exploration is needed.

## 5 Immunotherapy combined with antiangiogenic therapy in *EGFR* positive patients

### 5.1 The tumor angiogenesis and immune microenvironment in *EGFR* positive patients

Epidermal growth factor receptor (EGFR)-activating mutations represent the most frequent targetable alteration with a prevalence of nearly 20% in Caucasians with lung adenocarcinomas (79). Moreover, *EGFR* mutations in NSCLC have immunosuppressive effects, and previous studies have reported that the *EGFR* mutations can modulate several factors to impact TME, such as TILs, Tregs, MDSCs, TAMs, and immunoregulatory cytokines (80, 81). Preclinical studies have revealed that the VEGF and EGFR pathways share common downstream signaling, and these pathways can function exclusively of one another during oncogenesis (82). Moreover, in *EGFR*-mutant NSCLCs, *EGFR* activation may drive VEGF expression as *EGFR*-mutant NSCLC cells constitutively up-regulate HIF-1α in a hypoxia-independent manner (83). Therefore, mutant *EGFR*-driven NSCLC has a unique immunosuppressive microenvironment, and *EGFR* activation promotes the expression of VEGF, which is expected to result in tumor angiogenesis.

### 5.2 Clinical practices

According to NCCN guidelines, results of molecular testing including *ALK, BRAF, EGFR*, etc., should be obtained before administering first-line immunotherapy if clinically feasible. Patients with metastatic NSCLC and high PD-L1 expression level who also have a molecular variant that targets the driver oncogene should receive first-line targeted therapy rather than first-line immunotherapy because the former choice yields higher response rates and is better tolerated (39, 84). However, acquired resistance to targeted drugs often occurs in these patients, and there is usually no good treatment for the posterior line. As previously stated, *EGFR*-positive patients have a special immunosuppressive TME. What’s more, *EGFR* mutation can be associated with increased VEGF expression (85), and a sharing downstream signaling pathways of VEGF and EGFR was revealed (82), which brought us interest in the efficacy of anti-PD-1/PD-L1 antibodies plus antiangiogenetic therapy in *EGFR* positive patients.

In the subgroup analysis of Impower150, ABCP brought longer PFS among patients with *EGFR/ALK*-mutant than BCP (40, 86). The phase 3 ORIENT-31 research recruited 444 locally advanced or metastatic NSCLC patients with *EGFR*-mutant who progressed after previous *EGFR*-TKI treatment and categorized them into sintilimab + IBI305 (a biosimilar of bevacizumab) + pemetrexed + cisplatin group, sintilimab + pemetrexed + cisplatin group, and chemotherapy alone group. After a median follow-up of 9.8 months, the first group exhibited prolonged PFS relative to the chemotherapy alone group (6.9 months vs. 4.3 months, HR 0.46) (64). Another phase Ib/II clinical trial enrolled 43 advanced NSCLC patients with *EGFR+/ALK+* who had previously received targeted therapy. They received camrelizumab plus apatinib and showed a confirmed ORR of 18.6%, a clinical benefit response rate of 27.9%, and an mPFS of 2.8 months, assessed as moderate antitumor activity and acceptable safety profile (63). There was also research about uncommon *EGFR* mutations, enrolling 16 patients with EGFR mutations including Exon 18 (G719X, E709A), 20 insertion, 21 L861Q, and compound mutation and administrating atezolizumab + bevacizumab + carboplatin + (nab-)paclitaxel as first or further line treatment. ORR turned out to be 81.3%, disease control rate (DCR) was 87.5%, and mPFS reached 13.6 months (87). However, according to a meta-analysis evaluating the benefit-predicting factors of metastatic NSCLC, chemoimmunotherapy led to longer PFS regardless of *EGFR* or *ALK* status, whereas Impower130 exhibited no significant PFS superiority with non-bevacizumab-based regimens in *EGFR/ALK+* subgroups (88, 89).

There were more studies enrolling patients with EGFR or with other driver gene mutations, included in **Table 2**. Overall, the efficacy of this combined regimen in treated or untreated *EGFR+* patients has been proven, but further large-scale clinical trials are needed to confirm its prominent advantages.

## 6 Immunotherapy combined with antiangiogenic therapy in patients with distant organ metastases

Clinical trials have demonstrated the activity of immunotherapy in patients with brain and liver metastases (90, 91), and the activity was preliminarily confirmed by subgroup analyses of later clinical trials. The key subgroup analysis of Impower150 revealed a prolonged PFS in ABCP arm compared with BCP arm (13.2 months vs. 9.1 months) in liver metastatic ITT patients (92). Relatively prolonged mPFS was observed in patients with all bone, liver, and brain metastases in the phase III TASUKI-52 trial (46). However, brain metastasis could still be a significant risk factor for the combination therapy according to a phase Ib trial (55).

A retrospective study found the combination treatment of camrelizumab, apatinib, and radiotherapy was associated with lower levels of TRIM27, SCC-Ag, and CYFRA21-1, which might promote tumor infiltration, proliferation, and activation (67). As patients with oligometastases are in the transition from primary to extensive metastases, these patients will benefit from treatment effective for metastasis, which is worthy of further exploration.

**Table 2** lists most searchable clinical studies on the combination of immunotherapy and antiangiogenic therapy. Only prospective trials and retrospective studies with more than 50 participants were selected.

## 7 Predictive biomarkers of ICI plus antiangiogenetic treatment

Immunotherapy has changed the treatment landscape of NSCLC and has shown durable response rates in some refractory tumors. However, some treated patients show no response and serious immune-related side effects. Therefore, there is an urgent need for immunotherapeutic markers to help select people who are likely to benefit from immunotherapy, Multiple biomarkers have been explored over the years, and some emerging biomarkers are also under exploration (31). There are relatively mature studies of biomarkers separately in the immunotherapy field and antiangiogenetic field. However, few biomarkers focus specifically on the field of combination therapy. Here we summarize all common biomarkers, hoping to shed light on future treatments, especially in the field of combination therapy.

### 7.1 PD-L1 expression

Based on extensive research, ICI treatment plays a better role in immune inflamed tumors compared with noninflamed types (93). Tumors under immune inflamed conditions are characterized by high PD-L1 expression, high CD8+ T cell density, or strong IFN-γ cytolytic T cell signature (94). The expression of PD-L1 is the first established biomarker that can predict the clinical efficacy of PD-1/PD-L1 inhibitors, which has shown guiding significance in the treatment of many tumors, and the NCCN treatment guidelines recommend PD-L1 expression as a biomarker for immunotherapy (39). Most clinical studies of anti-PD-1/PD-L1 antibodies plus antiangiogenetic therapy included PD-L1 subgroup analysis. In Impower150, PFS in ABCP group was 8.0 months among patients with PD-L1<50% and 12.6 months for the subgroup with high PD-L1 expression (40). In another camrelizumab plus apatinib trial, a trend of better efficacy was observed in PD-L1 positive subgroup defined as TPS ≥1%, but without statistical significance (ORR 36.0% vs. 22.7%, P=0.20; mPFS 6.8 vs. 5.1 months, P=0.29) (54). Although PD-L1 expression is not the optimal biomarker, and its predictive role varies between different studies, which might be attributed to different assay methods, reagents sources, and selected thresholds, it is the best available biomarker to assess whether a patient is a suitable candidate in current clinical practice (39, 95, 96).

### 7.2 Tumor mutation burden

Tumor mutation burden (TMB), defined as the number of non-synonymous somatic mutations in cancer cells, is used to be another NCCN guideline recommended predictor in ICI therapy (39). Patients with high TMB levels tend to express high neoantigen levels on the surface of tumor cells that will activate the killing effect of immune cells (39). In June 2020, the FDA approved pembrolizumab for patients with unresectable or metastatic solid tumors with high TMB levels (≥10muts/Mb) that had progressed after prior therapy (97), which represented FDA acceptance of TMB as an independent biomarker for immunotherapy. However, there is a lack of consensus on the predictive value of TMB in clinical research results (98), and the lack of agreement on cut-off value and measurement standardization in clinical application led to its removal from the recommended immune biomarker by the NCCN panel in 2020 (99). In a phase Ib trial of 22 NSCLC patients, sintilimab plus anlotinib exhibited an ORR showing no significant association with TMB level (43).

Blood-based TMB (bTMB) provides a new way to measure TMB in patients who are difficult to obtain tumor tissue samples. Since high bTMB level can predict PFS benefit but not OS benefit in previous studies, Wang et al. proposed the concept of low allele frequence-bTMB (LAF-bTMB) and verified the prediction efficiency of LAF-bTMB algorithm through several international clinical cohorts (100). However, the predictor appears to have limited value at times. In a pilot study, bTMB could not stratify the PFS among 22 cases of second or further line camrelizumab plus apatinib treatment (95).

### 7.3 VEGF and ANG-2

Solid predictive biomarkers for VEGF signaling pathway targeted treatment have not been identified in tumor patients, whereas VEGF and angiopoietins are the key targets in related therapies (18). In the field of NSCLC, ANG-2, and βFGF was found to contribute to worse prognosis (101, 102), and higher plasma VEGF level might predict a better response to bevacizumab (102). VEGF-related molecules need to be further investigated as a potential predictive biomarker as data in response to anti-VEGF therapy or antiangiogenetic combination therapy are scarce.

### 7.4 Mutations in specific genes

Tumor driver genes can affect the tumor immune microenvironment and the response of immunotherapy. For example, *KRAS* and *TP53* mutated populations tend to express higher levels of PD-L1, and the combined treatment of ABCP led to OS improvements in patients with *KRAS* and *TP53* co-mutations in a subgroup analysis of Impower150. On the contrary, in *KRAS*, *STK11*, and *KEAP1* co-mutated patients, the expression of PD-L1 was reduced, and no apparent OS improvement was observed (103). However, the addition of apatinib to camrelizumab was reported to have better clinical outcomes in patients with mutation of *STK11* and/or *KEAP1* in another research, which indicated that the addition of antiangiogenetic drugs to ICI might also bring reverse effect against mono-agent immunotherapy (54, 104). The underlying reason might be attributed to the involvement of *STK11* and *KEAP1* in angiogenesis.

### 7.5 Liquid biopsy biomarkers

Other circulating markers such as circulating tumor DNA (ctDNA), circulating tumor cells (CTCs), exosomes, and circulating immune cells have also attracted research interest as surrogate markers of tumor burden (94). The concentration of ctDNA was identified as one of the independent risk factors of camrelizumab plus apatinib therapy in a multivariate Cox regression analysis, while ctDNA mutational status indicated a trend of difference but was not independent (95). Although many studies have explored the predictive value of liquid biopsy in immunotherapy, there is less exploration of combination therapy.

### 7.6 Others

There are also other genes, proteins, cells, or indexes that describe their status that have been proposed as biomarkers in mono-agent immunotherapy, including TIL, MSI-H/dMMR, CTLA4 expression, diversity of gut microbiome, etc. (94, 96). But rarely were reported in the field of combination immunotherapy. It also appears that no anti-PD-1/PD-L1 antibody plus antiangiogenetic specific biomarker have been identified. Further research on predictive biomarkers will be necessary.

## 8 Conclusion

In summary, a series of clinical studies have shown that anti-angiogenic drugs and ICIs have synergistic anti-tumor effects. On the one hand, anti-angiogenic drugs can reverse the immunosuppressive state of TME and enhance the efficacy of ICIs through immune reprogramming. On the other hand, ICIs can restore the immunosupportive microenvironment, promote vascular normalization, and enhance the efficacy of anti-angiogenic drugs. ICIs combined with antiangiogenic drugs have been widely and increasingly prescribed, but they also have drug resistance leading to treatment failure, and vascular normalization has a short specific time window. Therefore, how to delay the generation of drug-resistant cells is one of the urgent problems to be solved at present. Additionally, how to extend the normalization window effectively and explore the administration time, sequence, and optimal dosage of each drug in combination therapy may become the key for further research. Last but not least, how to select the most appropriate treatment plan for different patients to achieve the purpose of precise personalized treatment is of great importance, so it is also the direction of future research to find relevant biomarkers to predict the efficacy of patients and identify potential benefit groups. To sum up, more in-depth basic experiments and clinical studies are needed to optimize the treatment regimen of ICIs combined with anti-angiogenic drugs, so as to further improve patient prognosis with NSCLC.

## Author contributions

DH: Writing – original draft, Writing – review & editing. LW: Writing – original draft, Writing – review & editing. JX: Writing – review & editing. JZ: Writing – review & editing. HB: Writing – review & editing. JW: Funding acquisition, Writing – review & editing. 
